# Regional gas exchange evaluation during ex vivo lung perfusion in a swine model of localized lung dysfunction

**DOI:** 10.1002/ame2.70224

**Published:** 2026-06-15

**Authors:** Giulia Maria Ruggeri, Elena Chiodaroli, Matteo Montoli, Sara Pieropan, Alessandro Santini, Gianluca Lopez, Michele Battistin, Luigi Vivona, Sebastiano Maria Colombo, Jacopo Fumagalli, Eleonora Carlesso, Stefano Ferrero, Lorenzo Rosso, Stefano Gatti, Antonio Maria Pesenti, Giacomo Grasselli, Alberto Zanella

**Affiliations:** ^1^ Department of Anesthesia and Intensive Care Units ‘de Gasperis’ Cardio Center ASST Grande Ospedale Metropolitano Niguarda Milan Italy; ^2^ Department of Pathophysiology and Transplantation University of Milan Milan Italy; ^3^ Department of Anesthesia and Intensive Care ASST Santi Paolo e Carlo, San Paolo University Hospital Milan Italy; ^4^ Department of Thoracic Surgery ASST Santi Paolo e Carlo, San Paolo Hospital Milan Italy; ^5^ Department of Anesthesia and Intensive Care Units IRCCS Humanitas Research Hospital Milan Italy; ^6^ Pathology Unit Fondazione IRCCS Ca' Granda‐Ospedale Maggiore Policlinico Milan Italy; ^7^ Department of Biomedical, Surgical and Dental Sciences University of Milan Milan Italy; ^8^ Center for Preclinical Research Fondazione IRCCS Ca' Granda‐Ospedale Maggiore Policlinico Milan Italy; ^9^ Department of Anaesthesia and Intensive Care IRCCS MultiMedica San Giuseppe Hospital Milan Italy; ^10^ Anesthesia and Intensive Care for Adults Fondazione IRCCS Ca' Granda, Ospedale Maggiore Policlinico Milan Italy; ^11^ Department of Thoracic Surgery Fondazione IRCCS Ca' Granda Ospedale Maggiore Policlinico Milan Italy

**Keywords:** ex vivo lung perfusion, experimental swine model, lung morphology, lung transplantation, pulmonary gas exchange

## Abstract

**Background:**

Ex vivo lung perfusion (EVLP) allows the evaluation of lungs that do not meet standard transplantation criteria. Current procedures do not permit the identification of regional functional deficits. We investigated the feasibility of assessing lobar gas exchange during EVLP in a swine model.

**Methods:**

In five healthy swine, lungs were procured with beating heart and connected to an open‐atrium EVLP circuit. Each pulmonary vein was cannulated for lobar perfusate sampling. After baseline assessment, the left inferior bronchus (LIB) and then the left superior bronchus (LSB) were temporarily clamped to simulate localized injury. At each step, perfusate gas analyses from each vein and the left atrium (LA), lung mechanics, pulmonary hemodynamics, and computed tomography (CT) were obtained.

**Results:**

At baseline, no differences were observed between LA and lobar samples. Occlusion of LIB and LSB produced up to 95% non‐ or poorly aerated tissue in the affected lobe, with lobar shunt increasing to 97% [88%–103%] (LIB) and 97% [78%–106%] (LSB). Corresponding lobar PaO_2_/FiO_2_ fell to 47 [46–54] and 45 [43–67] mmHg, whereas LA‐mixed venous blood maintained higher values (299 [188–373] and 349 [347–377] mmHg, respectively). Bronchial occlusions caused overall significant worsening of lung function, including increased pulmonary vascular resistance, and reduction in total air lung volume and normally aerated tissue on CT scan analysis. In Left Inferior Lobe (LIL), histological samples showed significant interstitial congestion and alveolar hemorrhage.

**Conclusions:**

Lobar‐specific perfusate gas analysis during EVLP provides accurate functional assessment and enables the localization of lung injury, potentially improving graft evaluation before transplantation.

## Graphical abstract

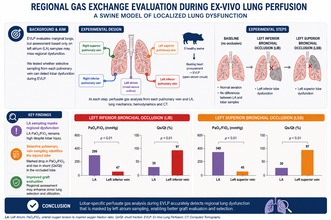

## 1 INTRODUCTION

Ex vivo lung perfusion (EVLP) is a well‐established technique used clinically to evaluate and recondition lungs that are not immediately suitable for transplantation.^1^ EVLP allows increasing the number of organs available for transplantation while achieving similar outcomes in recipients transplanted with standard lung grafts.^2^

Despite different techniques being adopted worldwide, mainly diverging in perfusate composition, flow, left atrium management, and ventilatory setting,^3^, ^4^, ^5^ the main purpose of EVLP is to assess lung function in a highly standardized setup.

During the standard 4–6 h of ex vivo perfusion, gas exchange, lung mechanics, and hemodynamics are monitored to assess lung suitability for transplantation. The lung gas exchange function, a key parameter in graft evaluation, is primarily determined, among all techniques, by assessing oxygenation in perfusate samples from the left atrium (LA), reflecting the global performance of the graft.^6^

However, localized lung damage or atelectasis, which is often present and detected by inspection or imaging techniques, is difficult to quantify and may lead to discarding grafts presenting only local dysfunction, which may be reversible, or accepting grafts with severe local dysfunction, possibly optimizable during EVLP.^7^

We hypothesized that blood gas analyses (BGA) of perfusate sampled from each pulmonary vein could confirm and quantify regional lung disfunction.

The aim of the present study was to demonstrate, in a preclinical model of lobar atelectasis following bronchial clamping, the role of regional gas exchange assessment in identifying areas of lung dysfunction during EVLP.

## 2 MATERIALS AND METHODS

Five healthy domestic female pigs (44 ± 2.7 kg) were used for the experiments. The Italian Ministry of Health approved the study (DL 26/2014), and animals were treated in conformity to international animal care recommendations.^8^

**FIGURE 1:**
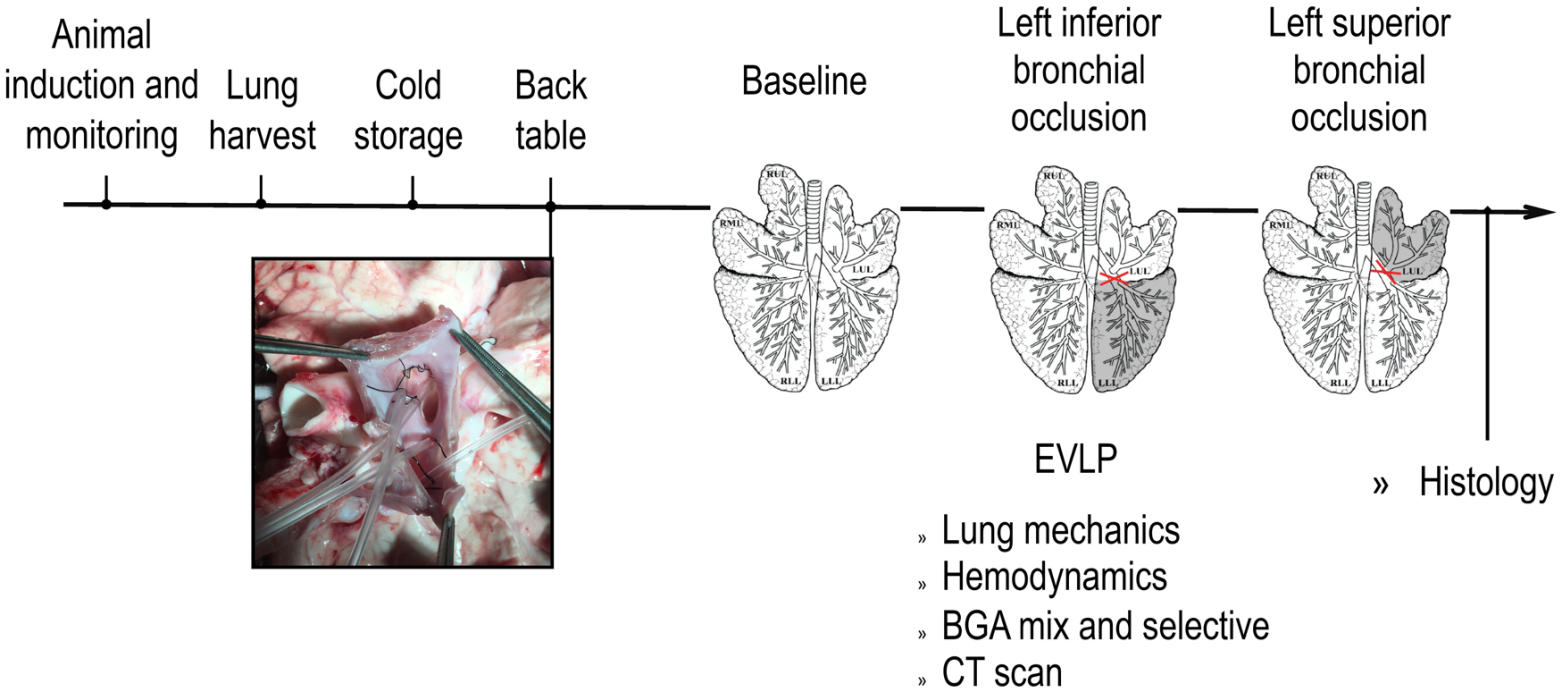
Experiment timeline and EVLP protocol. The picture in the square shows the cannulation of each pulmonary vein with the distal portion of a Nelaton catheter to perform selective BGA. BGA, blood gas analysis; CT, computer tomography; EVLP, ex vivo lung perfusion.

Figure 1 shows the timeline of the experiments. Preanesthesia was performed with an intramuscular injection of 1 mg medetomidine (Dormitor, Pfizer Animal Health, Exton, PA, USA) and 200 mg tiletamine hydrochloride (Zoletil, Virbac Srl, Milan, Italy). Anesthesia was induced with 20 mg propofol (Diprivan, AstraZeneca, Milan, Italy), and the airways were secured using a 7.0‐mm endotracheal tube. Total continuous intravenous (iv) anesthesia was maintained with propofol 4–12 mg/kg/h and medetomidine 1.25–5 μg/kg/h. Pancuronium bromide (Pancuronium Inresa, Inresa Arzneimittel GmbH, Freiburg, Germany) bolus 0.1 mg/kg iv and then continuous infusion 0.1–0.2 mg/kg/h were used for neuromuscular relaxation. Antibiotic prophylaxis with ceftriaxone 1 g (TEVA Italia, Milan, Italy) was administered. Animals were mechanically ventilated (Servo‐i Ventilator, Maquet Critical Care AB, Getinge Group, Sweden) in volume‐controlled mode with a tidal volume (TV) of 8 mL/kg, positive end‐expiratory pressure (PEEP) 5 cmH_2_O, fraction of inspired oxygen (FiO_2_) 40%, inspiratory‐to‐expiratory time ratio (I:E) 0.33, and respiratory rate (RR) 18–20 breaths/min set to maintain normocapnia (end‐tidal CO_2_ [EtCO_2_] target ~40 mmHg). Electrocardiogram, pulse oximetry, and an internal temperature probe were used for monitoring animal life parameters. A peripheral venous catheter was used for infusion of fluids with balanced solutions.

A median sternotomy was performed and, after systemic heparinization (300 IU/kg), an 18‐Fr cannula was inserted into the inferior vena cava to withdraw 1 L of blood into a collection bag previously prepared with 4000 IU unfractionated sodium heparin (Medic Italia S.r.l., Milan, Italy), 1‐g ceftriaxone, and 70‐mL acid‐citrate‐dextrose anticoagulant (ACD, Fresenius Kabi, Bad Hombrug, Germany). The collected blood was not washed but was subsequently leukocyte‐depleted using a leukocyte reduction filter (RC filter, Haemonetics Manufacturing Inc., Covina, CA, USA), as in the Transmedics OCS system, and stored for subsequent EVLP. The heart–lung block harvest was performed as previously reported.^9^ Briefly, a bolus of 250‐μg alprostadil (Prostin, Pfizer, Puurs, Belgium) was injected into the main pulmonary artery (PA) after a 16‐Fr cannula was placed into the PA. Superior and inferior cava veins were ligated, the ascending aorta was clamped, the left atrial appendage was transected, and the PA was flushed from a height of 20 cm above the heart with 60 mL/kg of cold (4°C) dialysate solution (Multibic, Fresenius, Bad Homburg, Germany) and subsequently with 1 L of Perfadex solution (XVIVO Perfusion AB, Göteborg, Sweden) activated with calcium chloride (60 mg), sodium nitroprusside (10 mg), and NaHCO_3_ (8.4%, 10 mL). During lung procurement, topical cooling was achieved with cold saline pleural cavities lavage while bronchial temperature was monitored. The trachea was clamped and the lungs were kept inflated with 80% FiO_2_. After retrieval, the heart–lung block was placed in slush ice with Perfadex solution and stored at 4°C for 60–75 min.

On the back table, the heart was removed, leaving the LA open, and a retrograde flush was performed with 1 L of Multibic solution under gravity drainage. The left superior and inferior bronchi were isolated with a tourniquet^10^ to allow an independent and temporary occlusion of each left‐sided bronchus (Figure 1). To obtain selective perfusate samples, each pulmonary vein was cannulated with an 8‐Fr bronchoaspiration catheter, proximally cut and connected to a 5‐Fr peripheral venous cannula (Figure 1, square). Lastly, the PA cannulation was performed (XVIVO Perfusion AB, Göteborg, Sweden), and an endotracheal tube with 7.0 mm internal diameter was introduced in the trachea, maintaining the lungs inflated. A temperature probe was placed in the LA. The lungs were transferred to the EVLP system, composed of a lung chamber (XVIVO Perfusion AB, Göteborg, Sweden) connected through 0.375‐in heparin‐coated polyvinyl tubing to a reservoir, a centrifugal pump (Rotaflow, Maquet Getinge Group, Rastatt, Germany), a gas exchange membrane lung with an arterial clot filter (Oxygenator PMP, Maquet Getinge group, Rastatt, Germany), and a heat exchanger. We adopted a low‐flow, open LA, cellular perfusate EVLP model, as previously described.^11^, ^12^, ^13^ The EVLP circuit was primed with 1.5 L of a cellular perfusate containing 2 L of a buffered dextran‐containing extracellular‐type solution with an optimized colloid osmotic pressure (see online supplement for perfusion solution composition) and 1 L of previously collected autologous blood (target hematocrit 10%–15%).^14^ Sodium heparin (20 000 IU), ceftriaxone (1 g), and methylprednisolone (500 mg) were added to the perfusate. Hourly exchanges of perfusate were made to maintain optimal glucose and electrolyte levels. The blood flow was increased stepwise up to 40% of estimated cardiac output (100 mL/kg of the pig weight) with an LA temperature target of 37°C. At 32°C, a lung‐protective ventilatory strategy was gradually started: TV (4–7 mL/kg), PEEP (5 cmH_2_O), FiO_2_ (40%), I:E (0.33), RR (7 breaths/min). The membrane lung was ventilated with 5 L/min of air and 0.35 L/min of CO_2_.

The study protocol consisted of three phases: baseline, occlusion of the left inferior bronchus (LIB), and occlusion of the left superior bronchus (LSB). At each step, after aspiration of secretions and a recruitment maneuverer, the membrane lung gas flow was switched to 5 L/min of nitrogen and 0.35 L/min of CO_2_ to deoxygenate the perfusate, whereas FiO_2_ on the ventilator was set to 100%. Lung mechanics and hemodynamic parameters were recorded, and a computed tomography (CT) scan was performed at end expiration. To evaluate global and regional gas exchange, six BGAs were performed simultaneously from the PA, LA, and the four pulmonary veins.^15^ To quantify the anatomical degree of lung disfunction after bronchial clamp, a quantitative analysis of the CT scan images was performed offline using dedicated software (Maluna 3.17, University Hospital of Göttingen, Germany). Lung parenchyma was partitioned according to density categories expressed in Hounsfield units (HU): overaerated (−1000 to −901 HU), normally aerated (−900 to −501 HU), poorly aerated (−500 to −101 HU), and nonaerated (−100 to +200 HU) tissue.^16^

**FIGURE 2:**
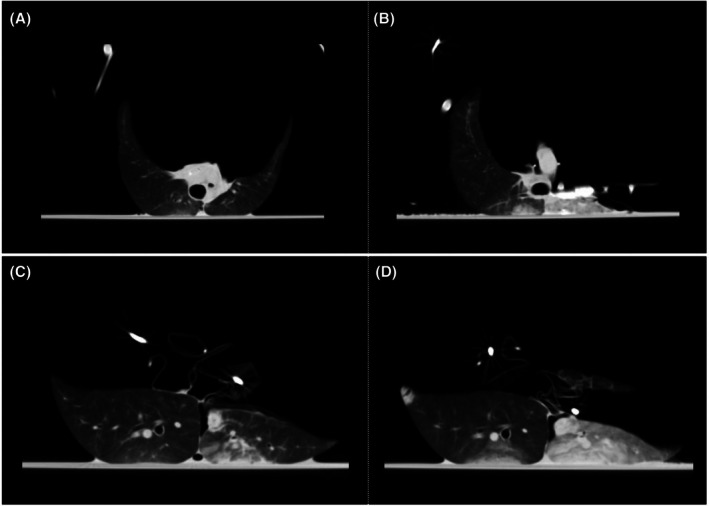
Comparison between the same portion of the lung at the computer tomography (CT) scan analysis at baseline (A) and during left superior bronchial occlusion (B), and at baseline (C) compared to left inferior bronchial occlusion (D).

Every bronchial clamp procedure was preceded by disconnection of the lungs from the ventilator until maximal deflation was obtained; bronchial occlusion was achieved by pulling the tourniquet; finally a recruitment maneuverer was performed to verify the efficacy of bronchial occlusion and simultaneously reaerate the remaining pulmonary parenchyma,^17^ as shown in Figure 2. The total EVLP time was approximately 4 h on average. The protocol included an initial 60‐min phase of gradual reperfusion and ventilation during controlled rewarming, after which baseline conditions were defined. Baseline measurements and every occlusion procedure lasted 60 min.

This study was designed as a preclinical physiological feasibility study aimed at evaluating the ability of selective pulmonary vein sampling to detect regional gas exchange impairment during EVLP. Because the induced intervention (lobar bronchial occlusion) produces large and deterministic physiological effects, and each animal serves as its own control in a repeated‐measures design, no formal sample‐size calculation was performed.

Data are expressed as median and interquartile range (IQR) because of the small sample size (*n* = 5). Before modeling, all variables were rank‐transformed to allow robust mixed‐effects analysis without requiring the assumption of normality. Rank‐based linear mixed‐effects models (PROC MIXED, SAS Institute) were then fitted, with pig included as a random effect and experimental phase or lung region as fixed effects, as appropriate. This approach is commonly used in small‐sample physiological studies, as it preserves the flexibility of mixed models while ensuring nonparametric robustness. Pairwise comparisons were obtained using Tukey correction, and a two‐sided *p* < 0.05 was considered statistically significant. Data were analyzed using SAS 9.4 (SAS Institute, Cary, NC, USA).

## 3 RESULTS

The timing of each phase of harvest and cold ischemia is reported in Table S1. The ventilatory and EVLP settings, along with pulmonary and hemodynamic findings throughout the different phases of the experiment, are shown in Table 1. Bronchial occlusions resulted in a significant deterioration of lung function, evidenced by a decrease in total air lung volume, as well as normally aerated tissue, and an increase in pulmonary vascular resistance (Table 1).

**TABLE 1:** Ventilatory, hemodynamic, computed tomography (CT) scan, and ex vivo lung perfusion (EVLP) parameters throughout the different phases of the experiment.

	Baseline	Left inferior bronchial occlusion	Left superior bronchial occlusion	*p*‐value
Tidal volume, mL/kg	7.0 [6.9–7.2]	7.0 [6.9–7.2]	7.0 [6.9–7.3]	0.300
Peak airway pressure, cmH_2_O	18 [15.9–18.7]	20.0 [19.3–21.7]	17.6 [17.1–21.8]	0.128
Plateau airway pressure, cmH_2_O	14.7 [12.9–15.0]	16.0 [15.5–17.2]	14.3 [14.2–18.0]	0.121
PEEP tot, cmH_2_O	5.9 [5.8–5.9]	5.9 [5.3–6.4]	5.8 [5.5–6.2]	0.720
Lung compliance, mL/cmH_2_O	39.8 [36.5–40.0]	30.8 [29.7–31.1]	35.0 [29.7–35.7]	0.098
EtCO_2_, mmHg	26.0 [22.0–26.0]	25.0 [22.0–25.0]	26.0 [21.0–27.0]	0.927
Vd/Vt, %	0.7 [0.7–0.8]	0.7 [0.7–0.8]	0.8 [0.7–0.8]	0.312
Blood flow, L/min	1.8 [1.8–1.9]	1.8 [1.8–1.8]	1.8 [1.8–1.8]	0.203
PAPm, mmHg	7.8 [7.6–8.0]	16.0 [15.1–19.5]**	16.0 [15.3–18.0]**	0.003
PVR, dyn*s/cm^5^	353.5 [335.5–362.5]	744.2 [663.7–871.5]**	744.2 [625.6–809]**	0.002
*T*, °C	36.5 [36.3–36.5]	36.0 [36.0–36.2]	36.4 [36.0–36.4]	0.070
Total lung air volume, mL	1169.9 [1165.1–1281.2]	836.6 [728.8–1051.3]**	880.8 [852.7–1011.5]**	0.001
Total lung tissue weight, g	555.2 [435.2–568.5]	600 [459–625.9]	641.5 [503.6–698.7]	0.079
Total lung overaerated, %	0.8 [0.8–1.2]	0.6 [0.5–0.8]	0.4 [0.4–0.4]*	0.016
Total lung normally aerated, %	68.2 [64.2–72.6]	45.9 [41.5–47.2]*	39.8 [35.2–45.2]**	0.007
Total lung poorly aerated, %	22 [19–26]	38.6 [35.5–38.6]**	37 [35.5–41.1]**	0.003
Total lung nonaerated, %	8.9 [7.3–9.3]	16.5 [14.7–19]*	18.9 [18.7–22.7]**	0.006

Abbreviations: EtCO_2_, end‐tidal CO_2_; PAPm, mean pulmonary artery pressure; PEEPtot, total positive end‐expiratory pressure; PVR, pulmonary vascular resistance; *T*, temperature; Vd/Vt, dead space to tidal volume ratio.

* *p* < 0.05 versus baseline.

** *p* < 0.01 versus baseline.

**FIGURE 3:**
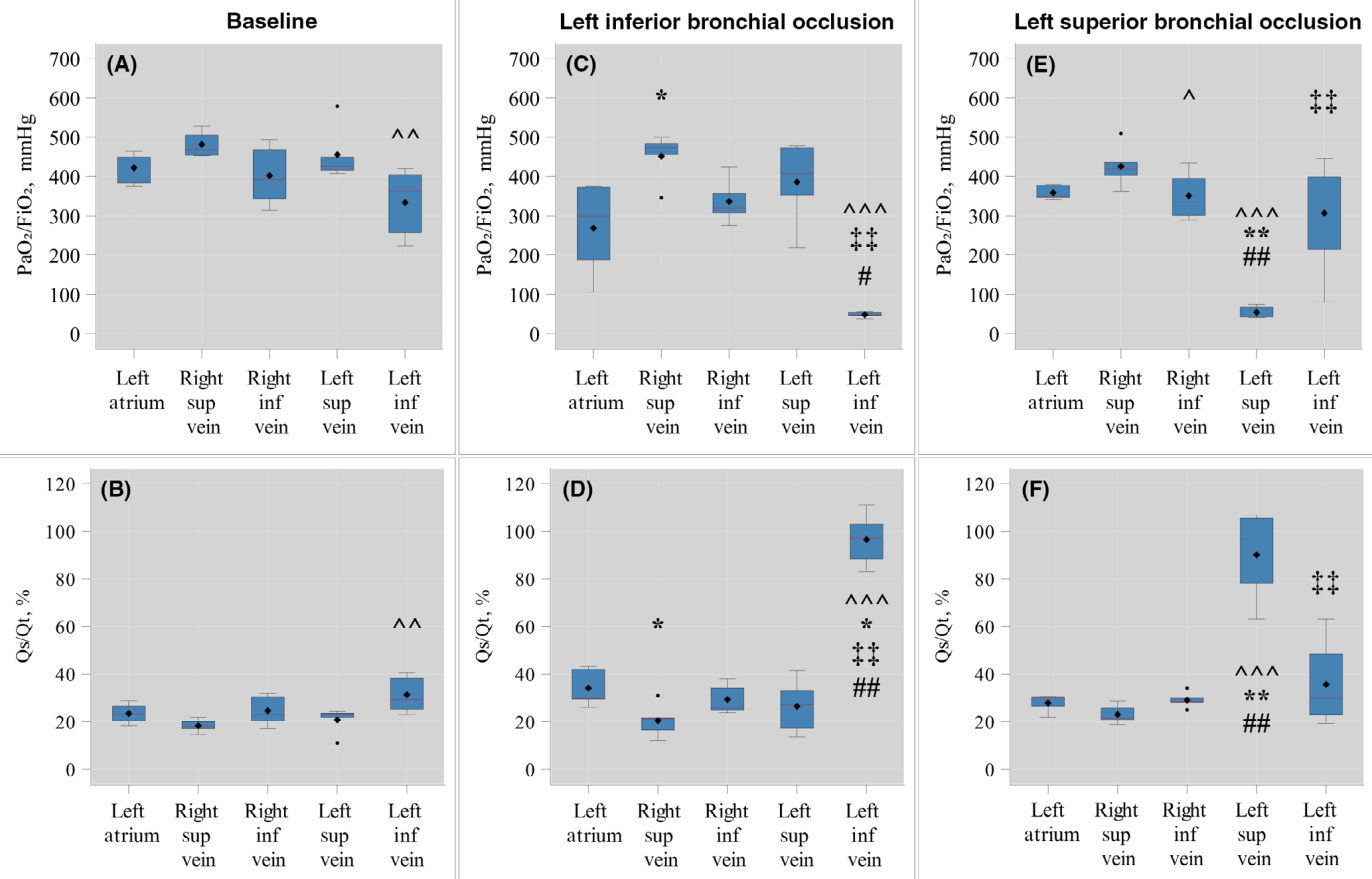
Lung oxygenation and anatomical shunt at baseline and during bronchial occlusions. Boxplots illustrate the distribution of PaO_2_/FiO_2_ ratio and pulmonary shunt fraction (Qs/Qt) across baseline conditions and selective bronchial occlusions. PaO_2_/FiO_2_: Arterial oxygen partial pressure to fractional inspired oxygen ratio; Qs/Qt: Pulmonary shunt fraction. Each boxplot displays the median (red horizontal line) and the interquartile range (IQR), spanning from the 25th to the 75th percentile. The whiskers extend to the most extreme values within 1.5 × IQR, and points beyond this threshold are plotted as outliers (black dots). The mean values are represented by diamonds. (A) PaO_2_/FiO_2_ at baseline. (B) PaO_2_/FiO_2_ during left inferior bronchial occlusion. (C) PaO_2_/FiO_2_ during left superior bronchial occlusion. (D) Qs/Qt at baseline. (E) Qs/Qt during left inferior bronchial occlusion. (F) Qs/Qt during left superior bronchial occlusion. *p < 0.05 versus left atrium, ^*p* < 0.05 versus right superior vein, ^#^ *p* < 0.05 versus right inferior vein, ^$^ *p* < 0.05 versus left inferior vein, **p < 0.01 versus left atrium, ^^*p* < 0.01 versus right superior vein, ^##^ *p* < 0.01 versus right inferior vein, ^‡‡^ *p* < 0.01 versus left superior vein, ^^^*p* < 0.001 versus right superior vein.

**FIGURE 4:**
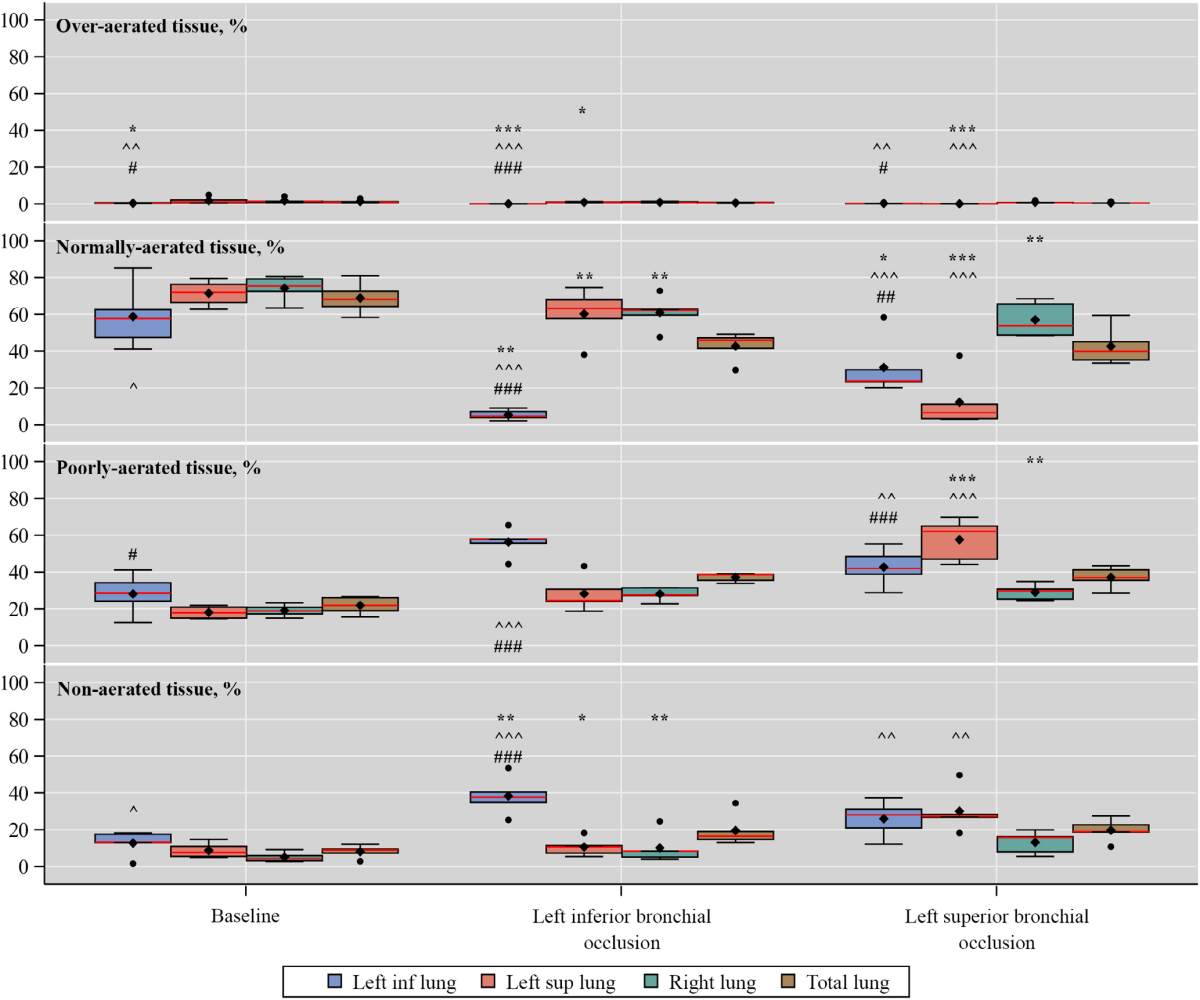
Computed tomography (CT) scan findings in the different lobes and in the total lung during the three phases of the experiment. Each boxplot displays the median (red horizontal line) and the interquartile range (IQR), spanning from the 25th to the 75th percentile. The whiskers extend to the most extreme values within 1.5 × IQR, and points beyond this threshold are plotted as outliers (black dots). The mean values are represented by diamonds. *p* < 0.05 is significant; *p < 0.05 versus total lung, ^*p* < 0.05 versus right lung, ^#^ *p* < 0.05 versus left superior lung, **p < 0.01 versus total lung, ^^*p* < 0.01 versus right lung, ^##^ *p* < 0.05 versus left superior lung, ***p < 0.001 versus total lung, ^^^*p* < 0.001 versus right lung, ^###^ *p* < 0.01 versus left superior lung.

At baseline, the LA PaO_2_/FiO_2_ was 439 [383–449] mmHg corresponding to an intrapulmonary shunt of 23% [20%–27%], with differences observed only in the contribution of left inferior and right superior pulmonary veins (Figure 3A,D). CT analysis shows a physiological aeration distribution with a minimal nonaerated component for both the left and right lungs (Figure 4).

After the LIB occlusion, a significant decrease in left inferior lobe gas volume from 251.9 [243.4–268.6] to 55.7 [50.7–73.8] mL (78% reduction, *p* < 0.001) was observed at the CT scan analysis, corresponding to an increase in poorly plus nonaerated tissue from 42% [37%–52%] to 95% [93%–96%] (*p* < 0.001). The overall lung oxygenation capacity significantly dropped with an LA PaO_2_/FiO_2_ of 299 [188–373] mmHg (*p* < 0.01 vs. baseline) and an LA Qs/Qt of 30% [29%–42%], not significantly different from baseline. Regional analysis revealed that only the left inferior lobe showed a significant increase in shunt fraction (97% [88%–103%], *p* < 0.001 vs. baseline) with an extremely low PaO_2_/FiO_2_ of 47 [46–54] mmHg (Figure 3B,E and Table S2). The left superior lobe and the right lung showed no alteration in aeration distribution or gas exchange.

Similarly, the LSB occlusion caused a significant decrease in LSL gas volume from 188.4 [181.1–191.4] to 32.4 [27.8–36.3] mL (83% reduction, *p* < 0.001 vs. baseline) with an increase in poorly plus nonaerated tissue 26% [23%–29%] to 93% [89%–97%] (*p* < 0.001). The LA PaO_2_/FiO_2_ was 349 [347–377] mmHg (*p* < 0.05 vs. baseline) with an LA Qs/Qt of 30% [27%–30%], not significantly different compared to baseline. Only the left superior lobe showed significant alteration with a regional shunt of 97% [78%–106%] (*p* < 0.01 vs. baseline), resulting in a PaO_2_/FiO_2_ of 45 [43–67] mmHg (*p* < 0.01 vs. baseline) (Figure 3C,F and Table S2). The left inferior lobe and the right lung showed oxygenation capacity and aeration distribution similar to baseline. Additional data of PaO_2_/FiO_2_ ratio and Qs/Qt % are reported in the online Table S2, as well as quantitative CT scan data at baseline and after bronchial occlusions (Table S3).

At the histological analysis, interstitial congestion and alveolar hemorrhage were significantly represented in the left inferior lobe; see online Table S4.

## 4 DISCUSSION

The occlusion of bronchi of both inferior and superior left lobes resulted in an almost complete loss of aeration, leading to a severe deterioration of the oxygenation capacity of the corresponding lobe. The proposed EVLP technique, which implements selective BGA of each pulmonary vein, allowed the detection of regional alterations in oxygenation with greater accuracy compared to commonly applied single BGA of mixed blood from the LA. This approach could prevent erroneous judgments of lung function and ensure suitability for transplantation.

Over the past two decades, EVLP has proven to be valuable tool for assessing donor lungs with extended criteria, increasing the number of organs available for transplantation by approximately 15%–20%. Recipients' outcomes are comparable to those of recipients receiving standard grafts, according to various studies.^2^, ^18^ Our EVLP circuit has a low‐flow, open LA, cellular perfusate setup. We use a continuous perfusion flow corresponding to approximately 40% of the donor's estimated cardiac output to minimize endothelial shear stress, thereby reducing pulmonary edema formation and inflammatory injury, as previously shown.^1^, ^19^ A slight positive left atrial pressure (2–4 mmHg) is considered physiologically protective for the pulmonary vasculature during isolated lung perfusion.^20^, ^21^ In our open‐atrium procedures, we directly measured pulmonary vein pressures and consistently observed slightly positive values, closely resembling physiological conditions.^13^ Moreover, open‐atrium procedures allow easy access to selective pulmonary vein sampling, which is central to the aim of the present study. Finally, in a cellular perfusate, the presence of red blood cells improves microvascular dynamics, promotes capillary recruitment, and favors vascular distension, mechanisms that are considered protective for the lung, particularly in open‐atrium configurations.^21^, ^22^, ^23^, ^24^ Furthermore, EVLP represents an ideal setting to apply and evaluate the efficacy of possible therapeutic strategies to modulate ischemia–reperfusion injury or to treat possible sources of organ damage, such as infections.^25^, ^26^, ^27^, ^28^, ^29^, ^30^, ^31^, ^32^, ^33^, ^34^ Finally, the EVLP platform may have a potential role in the field of pulmonology, oncology, and thoracic surgery.^35^

In this context of continuously evolving technology, having a thorough understanding of pulmonary physiology in the ex vivo setting and optimizing lung function evaluation is mandatory. There is ongoing debate as to whether oxygenation criteria should be the primary parameter for assessing donor lung function.^6^, ^36^, ^37^ In the present study, we utilized a preclinical experimental setting with a highly reproducible study protocol to assess the role of selective pulmonary vein sampling in evaluating regional lung gas exchange function in healthy and injured lungs. Through left‐sided inferior and superior bronchial occlusion, we induced almost complete lobar atelectasis, as indicated by reductions in lung volume and increase in the percentage of poorly and nonaerated tissue on the CT scan. In both open‐ and closed‐atrium EVLP techniques,^9^, ^38^ the overall lung oxygenation capacity is evaluated by sampling blood directly from the LA outflow, which is a mixture of blood from different pulmonary regions.^39^ Furthermore, LA blood withdrawal in the open‐atrium technique is operator‐dependent, leading to less reproducible results compared to the close technique.

Our findings suggest that relying solely on mixed blood withdrawal from the LA for the assessment of lung suitability for transplantation can be misleading. An oxygenation value above the PaO_2_/FiO_2_ cutoff of 300 mmHg for lung acceptance may conceal significant deterioration in lung function due to a localized damage, as shown in the present study during LSB occlusion. Conversely, a PaO_2_/FiO_2_ below the cutoff may result in graft discharge even in the presence of a localized lung injury,^40^ whereas the majority of the parenchyma remains in excellent condition, as we observed during LIB occlusion.

However, in both injuries, the oxygenation parameter recorded at the LA underestimated the extent of the lung injury,^41^ as the increase in the intrapulmonary shunt ranged from 4% to 7%, whereas the reduction in aeration was about three times higher. These results may be explained by hypoxic pulmonary vasoconstriction (HPV), which reduces blood flow in poorly ventilated regions and leads to significant increases in vascular resistance.^42^ Interestingly, recent studies have shown that HPV variation over time could be a sensitive parameter for evaluating lung quality during prolonged EVLP procedures.^43^

In conjunction with the alterations in gas exchange and pulmonary vascular resistance, we observed a significant reduction in total lung compliance, indicative of regional injury, during both bronchial occlusions (24% and 22% in the LIB and LSB, respectively). This finding is consistent with previous research by Benazzo et al.,^44^ which emphasizes the importance of compliance as a sensitive parameter for assessing donor lung function.

We have proposed a simple technique for assessing regional lung function by inserting a small catheter into each pulmonary vein outflow. This technique proved highly accurate in detecting lobar dysfunction. Furthermore, it allows for repeatable assessments without necessitating frequent chamber opening, thereby preserving sterility. Our study has underscored the limitations of single LA sampling for evaluating graft function during EVLP. In clinical practice, the identification of localized lung dysfunction could trigger a more focused approach to monitoring and treatment, such as regional lung recruitment or resection.^13^, ^45^, ^46^

The study has some limitations. First, the small sample size limits statistical power and does not allow for extensive multivariable modeling. However, this study was conceived as a preclinical physiological feasibility investigation, and the lobar bronchial occlusion produced large, deterministic alterations in regional aeration and gas exchange. Because each animal served as its own control within a repeated‐measures design, only a limited number of animals were required to demonstrate the physiological signal of interest. Second, bronchial occlusions were performed only on the left side of the lung, and the effect of right lung bronchial ligation was not explored. This decision was made due to the complex bronchial tree anatomy in pigs, which makes it difficult to isolate the right lobes without causing damage to the surrounding tissues.^10^ Additionally, no randomization was applied to the bronchial occlusion sequence, and left inferior lung ligation was always performed first. This was done to prevent further deterioration of the already mild impaired left inferior lobe as proved by CT scan and selective BGA from left inferior vein (Figures 3 and 4). Despite being extremely useful technologies in this contest, lung ultrasound, near‐infrared fluorescence (NIRF) imaging, and multiparametric magnetic resonance imaging (MRI) are not available at our laboratory. Finally, no therapeutic interventions were utilized to demonstrate the restoration of oxygenation after lobar lung recruitment or surgical resection/lobar artery ligation of the damaged lobe. Future experiments should explore the potential benefits of such treatments.

## 5 CONCLUSIONS

In conclusion, the cannulation of pulmonary veins during EVLP with open‐atrium technique can lead to a more accurate assessment of pulmonary function, continuous monitoring, and prompt identification of potential lung injury without invasiveness. Therapeutic interventions, such as interventional bronchoscopy or surgical resection, could be employed to treat pulmonary damage, enabling the retrieval of otherwise discarded lungs and, consequently, increasing the pool of transplantable organs.

## AUTHOR CONTRIBUTIONS

**Giulia Maria Ruggeri:** Conceptualization; data curation; formal analysis; methodology; writing – original draft. **Elena Chiodaroli:** Data curation; formal analysis; investigation; methodology; writing – original draft. **Matteo Montoli:** Methodology; writing – original draft. **Sara Pieropan:** Investigation; methodology; writing – original draft. **Alessandro Santini:** Data curation; formal analysis; investigation; methodology; supervision; writing – original draft. **Gianluca Lopez:** Investigation; methodology; writing – original draft. **Michele Battistin:** Data curation; formal analysis; investigation; methodology; writing – original draft. **Luigi Vivona:** Investigation; writing – original draft. **Sebastiano Maria Colombo:** Conceptualization; data curation; formal analysis; investigation; methodology; writing – original draft; writing – review and editing. **Jacopo Fumagalli:** Investigation; writing – original draft. **Eleonora Carlesso:** Data curation; formal analysis; validation. **Stefano Ferrero:** Supervision; writing – original draft; writing – review and editing. **Lorenzo Rosso:** Supervision; validation; writing – original draft; writing – review and editing. **Stefano Gatti:** Supervision; validation; writing – review and editing. **Antonio Maria Pesenti:** Resources; supervision; validation; writing – review and editing. **Giacomo Grasselli:** Resources; supervision; validation; writing – review and editing. **Alberto Zanella:** Conceptualization; data curation; formal analysis; funding acquisition; investigation; methodology; project administration; resources; supervision; validation; writing – original draft; writing – review and editing.

## FUNDING INFORMATION

The study was supported by “5 × 1000” 2014, devolved to Fondazione IRCCS Ca′ Granda Ospedale Maggiore Policlinico “Improvement of perfusion of isolated organs before transplantation through perfusion fluid optimization” and “Implementation of strategies for laboratory animal care,” and by the Italian Ministry of Health—Current Research IRCCS.

## CONFLICT OF INTEREST STATEMENT

The authors certify that they have no affiliations with or involvement in any organization or entity with any financial or nonfinancial interest in the subject matter discussed in this manuscript.

## ETHICS STATEMENT

The Italian Ministry of Health approved the study (DL 26/2014), and animals were treated in accordance with international animal care recommendations.

## Supporting information


**Table S1:** Timing of procedures during the experiments. Data are reported as median and interquartile range (IQR).
**Table S2:** PaO_2_/FiO_2_ ratio (mmHg) [upper panel] and Qs/Qt % [lower panel] in blood samples from left atrium and each pulmonary vein throughout the different phases of the experiment. One‐way analysis of variance (ANOVA), *p* < 0.05 as significant, * versus baseline, # versus left inferior bronchial occlusion, ^ versus left superior bronchial occlusion.
**Table S2:** PaO_2_/FiO_2_ ratio (mmHg) [upper panel] and Qs/Qt % [lower panel] in blood samples from left atrium and each pulmonary vein throughout the different phases of the experiment. *p < 0.05 versus baseline; **p < 0.01 versus baseline; ***p < 0.001 versus baseline; #*p* < 0.05 versus left inferior bronchial occlusion; ##*p* < 0.01 versus left inferior bronchial occlusion; ^*p* < 0.05 versus left superior bronchial occlusion; ^^*p* < 0.01 versus left superior bronchial occlusion.
**Table S3:** Quantitative computed tomography (CT) scan data at baseline, after left inferior bronchial (Inf Br) occlusion, and after superior bronchial (Sup Br). Overall analysis of total lung and selective analysis of right and left superior and left inferior lung. Overaerated: −1000 to −901 Hounsfield unit (HU); normally aerated: −900 to −501 HU; poorly aerated: −500 to −101 HU; nonaerated: −100 to +200 HU. Data are expressed as mean ± standard deviation (SD), *p < 0.05 versus baseline, **p < 0.01 versus baseline, ***p < 0.001 versus baseline, ^§^ *p* < 0.05 versus left inferior bronchial occlusion, ^§§^ *p* < 0.01 versus left inferior bronchial occlusion, ^§§§^ *p* < 0.001 versus left inferior bronchial occlusion. Air volume, tissue weight, and density distribution were compared to proc. mixed on ranks on timing.
**Table S4:** Histological findings at the end of the experiment in the different lobes. Each pathological change in all lung lobes studied in every experiment was evaluated with a score ranging from 0 to 3, indicating different degrees of damage: 0, absence; 1, mild; 2, moderate; 3, severe. Proc mixed *n* ranks: *p < 0.05 versus right apex lobe; ^*p* < 0.05 versus right middle lobe.
