# Supplementary material for: Regional gas exchange evaluation during ex vivo lung perfusion in a swine model of localized lung dysfunction
Source: Animal Model Exp Med. 2026 Jun 15;9(8):1517–26. doi: 10.1002/ame2.70224 (PMC13394259; doi:10.1002/ame2.70224)
Supplement: Supplementary file 1 [file AME2-9-1517-s001.docx]

**Regional gas exchange evaluation during Ex-Vivo Lung Perfusion in a swine model of localized lung dysfunction**

Ruggeri Giulia Maria ^a,b^, Chiodaroli Elena ^b,c^, Montoli Matteo ^d^, Pieropan Sara ^b^, Santini Alessandro ^e^, Lopez Gianluca ^f,g^, Battistin Michele ^h^, Vivona Luigi ^i^, Colombo Sebastiano Maria ^b,j^, Fumagalli Jacopo ^j^, Eleonora Carlesso ^b^, Ferrero Stefano ^f,g^, Rosso Lorenzo ^b,k^, Gatti Stefano ^h^, Pesenti Antonio Maria ^b,j^, Grasselli Giacomo ^b, j^, Zanella Alberto ^b,j^.

*Electronic Supplementary material*

**Affiliations:**

^a^ Department of Anesthesia and Intensive Care Units "De Gasperis" Cardio Center, ASST Grande Ospedale Metropolitano Niguarda, Milan, Italy.

^b^ Department of Pathophysiology and Transplantation, University of Milan, Milan, Italy

^c^ Department of Anesthesia and Intensive Care, ASST Santi Paolo e Carlo, San Paolo University Hospital, Milan, Italy

^d^ Department of Thoracic Surgery, ASST Santi Paolo e Carlo, San Paolo Hospital, Milan, Italy

^e^ Department of Anesthesia and Intensive Care Units, IRCCS Humanitas Research Hospital, Rozzano, Milan, Italy.

^f^ Pathology Unit, Fondazione IRCCS Ca' Granda-Ospedale Maggiore Policlinico, Milan, Italy.

^g^ Department of Biomedical, Surgical and Dental Sciences, University of Milan

^h^ Center for Preclinical Research, Fondazione IRCCS Ca’ Granda-Ospedale Maggiore Policlinico, Milano, Italy

^i^ IRCCS MultiMedica San Giuseppe Hospital, Department of Anaesthesia and Intensive Care. Milan, Italy.

^j^ Anesthesia and Intensive Care for Adults, Fondazione IRCCS Ca' Granda, Ospedale Maggiore Policlinico, Milan, Italy

^k^ Department of Thoracic Surgery, Fondazione IRCCS Ca' Granda Ospedale Maggiore Policlinico, Milan, Italy

**Additional Methods:**

**Perfusion solution:**

The present protocol used an acellular perfusion fluid with extra-cellular electrolyte composition. The solution was freshly prepared in sterile conditions in a laminar flow hood, using: albumin, NaCl, Perfadex (XVIVO Perfusion AB, Göteborg, Sweden), amphotericin B, glucose, CaCl_2_, KCl, K_3_PO_4_, MgSO_4_, and heparin. Although two separate lots of albumin with different sodium concentrations were used, electrolyte composition was maintained stable. Characteristics of the two solutions were: osmolality 294 and 286 mOsm/kg; albumin 8.8 and 8.9 g/dl; total protein 9.4 g/ dl for both lots. The perfusion fluid was filtered using a vacuum filtration unit to remove any particulate impurity or bacterial contamination and stored for a maximum of 5 days to avoid bacterial growth. Before each experiment, the fluid was added with cefazolin and NaHCO_3_, whereas no treatment with corticosteroids or other anti-inflammatory drugs was performed.

**Additional Results:**

**Timing of procedures during the experiment**

| **Procedures** | | **Time (min)** |
| --- | --- | --- |
| **Harvest Total** |  | 99 [94 - 110 ] |
|  | **Harvest preflush** | 75 [66 - 85 ] |
|  | **Harvest postflush** | 26 [25 - 27 ] |
| **Cold ischemia Total** |  | 184 [167 - 187 ] |
|  | **Flushing** | 12 [11 - 12 ] |
|  | **Fridge** | 65 [61 - 70 ] |
|  | **Back Table** | 76 [74 - 96 ] |

Table S1: Timing of procedures during the experiments. Data are reported as median and [IQR].

**PaO_2_/FiO_2_ and Shunt fraction (Qs/Qt %) during the experiment**

Table S2: PaO_2_/FiO_2_ ratio (mmHg) [upper panel] and Qs/Qt % [lower panel] in blood samples from left atrium and each pulmonary vein throughout the different phases of the experiment. One Way ANOVA, p<0.05 as significant, * vs Baseline, # vs Left Inferior Bronchial Occlusion, ^ vs Left Superior Bronchial Occlusion.

|  |  | **Baseline** | **Left Inferior Bronchial Occlusion** | **Left Superior Bronchial Occlusion** | **p-value** |
| --- | --- | --- | --- | --- | --- |
| **PaO_2_/FiO_2_, mmHg** |  |  |  |  |  |
|  | **Left Atrium** | 439 [383 - 449] | 299 [188 - 373]** | 349 [347 - 377]* | 0.0051 |
|  | **Right Superior Vein** | 468 [454 - 505] | 472 [455 - 484] | 419 [402 - 436] | 0.1315 |
|  | **Right Inferior Vein** | 392 [344 - 468] | 319 [307 - 356] | 334 [301 - 395] | 0.1845 |
|  | **Left Superior Vein** | 426 [416 - 449] | 407 [353 - 472] | 45 [43 - 67]**## | 0.0015 |
|  | **Left Inferior Vein** | 363 [258 - 404] | 47 [46 - 54]***^^ | 350 [214 - 399] | 0.0008 |
| **Qs/Qt, %** |  |  |  |  |  |
|  | **Left Atrium** | 23 [20 - 27] | 30 [29 - 42] | 30 [27 - 30] | 0.0799 |
|  | **Right Superior Vein** | 18 [17 - 20] | 21 [16 - 21] | 21 [21 - 26] | 0.0584 |
|  | **Right Inferior Vein** | 23 [20 - 30] | 26 [25 - 34] | 28 [28 - 30] | 0.3508 |
|  | **Left Superior Vein** | 23 [22 - 24] | 27 [17 - 33] | 97 [78 - 106]**## | 0.0011 |
|  | **Left Inferior Vein** | 29 [25 - 38] | 97 [88 - 103]**^^ | 30 [23 - 48] | 0.0011 |

Table S2: PaO_2_/FiO_2_ ratio (mmHg) [upper panel] and Qs/Qt % [lower panel] in blood samples from left atrium and each pulmonary vein throughout the different phases of the experiment. * p<0.05 vs BASELINE; ** p<0.01 vs BASELINE; *** p<0.001 vs BASELINE; # p<0.05 vs Left Inferior Bronchial Occlusion; ## p<0.01 vs Left Inferior Bronchial Occlusion; ^ p<0.05 vs Left Superior Bronchial Occlusion; ^^ p<0.01 vs Left Superior Bronchial Occlusion

**Quantitative CT scan data at baseline and during the experiment**

| **A) Total Lung** | **Baseline** | **Left Inf Br Occlusion** | **Left Sup Br Occlusion** | **p-value** | **B) Right Lung** | **Baseline** | **Left Inf Br Occlusion** | **Left Sup Br Occlusion** | **p-value** |
| --- | --- | --- | --- | --- | --- | --- | --- | --- | --- |
| **Air volume, mL** | 1169.9 [1165.1 - 1281.2] | 836.6 [728.8 - 1051.3]** | 880.8 [852.7 - 1011.5]** | 0.001 | **Air volume, mL** | 738 [705.1 - 863.5] | 671.7 [558.4 - 772.5] | 678.1 [671.1 - 697.3] | 0.219 |
| **Tissue weight, g** | 555.2 [435.2 - 568.5] | 600 [459 - 625.9] | 641.5 [503.6 - 698.7] | 0.079 | **Tissue weight, g** | 308.2 [246.3 - 310.7] | 340.7 [248.5 - 375.7] | 377.5 [263.9 - 383]** | 0.006 |
| **Over-aerated, %** | 0.8 [0.8 - 1.2] | 0.6 [0.5 - 0.8] | 0.4 [0.4 - 0.4]* | 0.016 | **Over-aerated, %** | 1.3 [0.8 - 1.4] | 0.7 [0.7 - 1.1] | 0.6 [0.6 - 0.7] | 0.058 |
| **Normally-aerated, %** | 68.2 [64.2 - 72.6] | 45.9 [41.5 - 47.2]* | 39.8 [35.2 - 45.2]** | 0.007 | **Normally-aerated, %** | 75.4 [72.5 - 79.2] | 62.2 [59.6 - 62.7]* | 53.9 [48.8 - 65.5]* | 0.009 |
| **Poorly-aerated, %** | 22 [19 - 26] | 38.6 [35.5 - 38.6]** | 37 [35.5 - 41.1]** | 0.003 | **Poorly-aerated, %** | 18.8 [17.2 - 20.9] | 27.6 [27.3 - 31.4]** | 29.8 [25.3 - 30.7]** | 0.003 |
| **Non-aerated, %** | 8.9 [7.3 - 9.3] | 16.5 [14.7 - 19]* | 18.9 [18.7 - 22.7]** | 0.006 | **Non-aerated, %** | 4.5 [3.2 - 6] | 8.3 [5.2 - 8.4] | 15.9 [8 - 16.2] | 0.078 |
| **C) Left Superior Lung** | **Baseline** | **Left Inf Br Occlusion** | **Left Sup Br Occlusion** | **p-value** | **D) Left Inferior Lung** | **Baseline** | **Left Inf Br Occlusion** | **Left Sup Br Occlusion** | **p-value** |
| **Air volume, mL** | 188.4 [181.1 - 191.4] | 119.7 [113.6 - 192] | 32.4 [27.8 - 36.3]**§ | 0.003 | **Air volume, mL** | 251.9 [243.4 - 268.6] | 55.7 [50.7 - 73.8]*** | 178.3 [153.9 - 224.7]§ | 0.001 |
| **Tissue weight, g** | 72.8 [61.1 - 80.5] | 66.6 [53.1 - 88] | 75.7 [70.7 - 81.1] | 0.443 | **Tissue weight, g** | 155.4 [148 - 166.7] | 163 [159.8 - 192.7] | 187.8 [158.6 - 245.5] | 0.122 |
| **Over-aerated, %** | 0.9 [0.6 - 2.1] | 0.9 [0.7 - 1] | 0 [0 - 0.1]**§§ | 0.003 | **Over-aerated, %** | 0.3 [0.2 - 0.6] | 0 [0 - 0.1]** | 0.2 [0.1 - 0.2]§ | 0.003 |
| **Normally-aerated, %** | 72 [66.5 - 76.3] | 63.2 [57.7 - 67.9] | 6.6 [3.3 - 11.1]***§§ | 0.001 | **Normally-aerated, %** | 57.7 [47.3 - 62.6] | 4.7 [3.9 - 7.2]*** | 23.7 [23.5 - 29.9]§§ | 0.000 |
| **Poorly-aerated, %** | 17.8 [15 - 21] | 24.5 [24 - 30.6]* | 62.1 [47 - 65]***§§ | 0.000 | **Poorly-aerated, %** | 28.5 [24.1 - 34.2] | 57.7 [55.7 - 57.9]*** | 41.9 [38.9 - 48.4]§ | 0.001 |
| **Non-aerated, %** | 7.7 [5.5 - 10.9] | 10.7 [7.3 - 11.4] | 27.3 [26.8 - 28.3]**§§ | 0.002 | **Non-aerated, %** | 13.3 [13 - 17.5] | 37.6 [34.8 - 40.4]** | 28.1 [21 - 31.2] | 0.005 |

Table S3: Quantitative CT scan data at baseline, after left inferior bronchial (Inf Br) occlusion and after superior bronchial (Sup Br): Overall analysis of total lung and selective analysis of right, left superior and left inferior lung. Over-aerated: -1000 -901 HU, Hounsfield Unit; Normally-aerated -900 -501 HU; Poorly-aerated -500 -101 HU; Non-aerated -100 +200 HU. Data are expressed as mean±SD, * p<0.05 vs Baseline, ** p<0.01 vs Baseline, *** p<0.001 vs Baseline, § p<0.05 vs Left Inferior Bronchial Occlusion, §§ p<0.01 vs Left Inferior Bronchial Occlusion, §§§ p<0.001 vs vs Left Inferior Bronchial Occlusion. Air volume, tissue weight and density distribution were compared with proc mixed on ranks on timing.

**Histological findings at the end of the experiment in the different lobes.**

|  | **Right Apex** | **Right Medium** | **Right Base** | **Left Apex** | **Left Medium** | **Left Base** | **p-value** |
| --- | --- | --- | --- | --- | --- | --- | --- |
| **Emphysematous change** | 1 [1 - 1] | 1 [1 - 1] | 1 [1 - 1] | 1 [1 - 1] | 1 [1 - 1] | 1 [1 - 1] | Stopped because of infinite likelihood |
| **Interstitial congestion** | 1 [0 - 1] | 1 [1 - 1] | 3 [1 - 3] | 2 [2 - 2] | 2 [1 - 2] | 3 [2 - 3] | 0.0449 |
| **Alveolar hemorrhage** | 0 [0 - 1] | 0 [0 - 1] | 3 [0 - 3] | 2 [2 - 2] | 1 [1 - 2] | 3 [3 - 3]* | 0.0211 |
| **Alveolar neutrophil infiltration** | 1 [1 - 1] | 2 [1 - 2] | 1 [1 - 1] | 1 [1 - 2] | 2 [1 - 2] | 2 [1 - 2] | 0.2485 |
| **Alveolar macrophage proliferation** | 2 [2 - 2] | 2 [2 - 2] | 2 [2 - 3] | 2 [2 - 3] | 2 [2 - 2] | 3 [2 - 3] | 0.0894 |
| **Alveolar type II pneumocytres proliferation** | 1 [0 - 1] | 1 [1 - 1] | 1 [0 - 1] | 1 [1 - 1] | 1 [1 - 1] | 1 [1 - 1] | 0.229 |
| **Interstitial lymphocytes proliferation** | 2 [1 - 2] | 2 [2 - 2] | 2 [2 - 2] | 2 [2 - 2] | 2 [2 - 2] | 2 [2 - 2] | 0.4031 |
| **Interstitial thickening** | 1 [1 - 1] | 1 [1 - 1] | 1 [1 - 1] | 1 [1 - 1] | 1 [1 - 1] | 1 [1 - 1] | 0.2721 |
| **Hyaline membrane formation** | 1 [0 - 1] | 1 [1 - 1] | 1 [1 - 1] | 1 [1 - 1] | 1 [1 - 1] | 1 [1 - 1] | 0.3866 |
| **Interstitial fibrosis** | 0 [0 - 0] | 0 [0 - 0] | 0 [0 - 0] | 0 [0 - 0] | 0 [0 - 0] | 0 [0 - 0] | 0.5627 |
| **Organization of alveolar exudate** | 0 [0 - 0] | 0 [0 - 0] | 0 [0 - 0] | 0 [0 - 0] | 0 [0 - 0] | 0 [0 - 0] | 0.443 |
| **Total** | 10 [7 - 10] | 11 [10 - 11] | 13 [10 - 15] | 14 [13 - 16] | 13 [13 - 14] | 16 [15 - 17]*^ | 0.0211 |

Table S4: Histological findings at the end of the experiment in the different lobes. Each pathological change in all lung lobes studied of every experiment was evaluated with a score ranging 0-3 and indicating different degree of damage: 0, absence; 1, mild; 2, moderate; 3, severe. Proc mixed n ranks * p<0.05 vs right apex lobe; ^ p<0.05 vs right middle lobe.
