# Supplementary material for: Regional gas exchange evaluation during ex vivo lung perfusion in a swine model of localized lung dysfunction
Source: Animal Model Exp Med. 2026 Jun 15:10.1002/ame2.70224. Online ahead of print. doi: 10.1002/ame2.70224 (PMC13394259; doi:10.1002/ame2.70224)
Supplement: Supplementary file 1 — Table S1: Timing of procedures during the experiments. Data are reported as median and interquartile range (IQR). Table S2: PaO2/FiO2 ratio (mmHg) [upper panel] and Qs/Qt % [lower panel] in blood samples from left atrium and each pulmonary vein throughout the different phases of the experiment. One‐way analysis of variance (ANOVA), p < 0.05 as significant, * versus baseline, # versus left inferior bronchial occlusion, ^ versus left superior bronchial occlusion. Table S2: PaO2/FiO2 ratio (mmHg) [upper panel] and Qs/Qt % [lower panel] in blood samples from left atrium and each pulmonary vein throughout the different phases of the experiment. *p < 0.05 versus baseline; **p < 0.01 versus baseline; ***p < 0.001 versus baseline; #p < 0.05 versus left inferior bronchial occlusion; ##p < 0.01 versus left inferior bronchial occlusion; ^p < 0.05 versus left superior bronchial occlusion; ^^p < 0.01 versus left superior bronchial occlusion. Table S3: Quantitative computed tomography (CT) scan data at baseline, after left inferior bronchial (Inf Br) occlusion, and after superior bronchial (Sup Br). Overall analysis of total lung and selective analysis of right and left superior and left inferior lung. Overaerated: −1000 to −901 Hounsfield unit (HU); normally aerated: −900 to −501 HU; poorly aerated: −500 to −101 HU; nonaerated: −100 to +200 HU. Data are expressed as mean ± standard deviation (SD), *p < 0.05 versus baseline, **p < 0.01 versus baseline, ***p < 0.001 versus baseline, § p < 0.05 versus left inferior bronchial occlusion, §§ p < 0.01 versus left inferior bronchial occlusion, §§§ p < 0.001 versus left inferior bronchial occlusion. Air volume, tissue weight, and density distribution were compared to proc. mixed on ranks on timing. Table S4: Histological findings at the end of the experiment in the different lobes. Each pathological change in all lung lobes studied in every experiment was evaluated with a score ranging from 0 to 3, indicating different degrees of damage: [file AME2-9999-0-s001.docx]

**Regional gas exchange evaluation during Ex-Vivo Lung Perfusion in a swine model of localized lung dysfunction**

Ruggeri Giulia Maria ^a,b^, Chiodaroli Elena ^b,c^, Montoli Matteo ^d^, Pieropan Sara ^b^, Santini Alessandro ^e^, Lopez Gianluca ^f,g^, Battistin Michele ^h^, Vivona Luigi ^i^, Colombo Sebastiano Maria ^b,j^, Fumagalli Jacopo ^j^, Eleonora Carlesso ^b^, Ferrero Stefano ^f,g^, Rosso Lorenzo ^b,k^, Gatti Stefano ^h^, Pesenti Antonio Maria ^b,j^, Grasselli Giacomo ^b, j^, Zanella Alberto ^b,j^.

*Electronic Supplementary material*

**Affiliations:**

^a^ Department of Anesthesia and Intensive Care Units "De Gasperis" Cardio Center, ASST Grande Ospedale Metropolitano Niguarda, Milan, Italy.

^b^ Department of Pathophysiology and Transplantation, University of Milan, Milan, Italy

^c^ Department of Anesthesia and Intensive Care, ASST Santi Paolo e Carlo, San Paolo University Hospital, Milan, Italy

^d^ Department of Thoracic Surgery, ASST Santi Paolo e Carlo, San Paolo Hospital, Milan, Italy

^e^ Department of Anesthesia and Intensive Care Units, IRCCS Humanitas Research Hospital, Rozzano, Milan, Italy.

^f^ Pathology Unit, Fondazione IRCCS Ca' Granda-Ospedale Maggiore Policlinico, Milan, Italy.

^g^ Department of Biomedical, Surgical and Dental Sciences, University of Milan

^h^ Center for Preclinical Research, Fondazione IRCCS Ca’ Granda-Ospedale Maggiore Policlinico, Milano, Italy

^i^ IRCCS MultiMedica San Giuseppe Hospital, Department of Anaesthesia and Intensive Care. Milan, Italy.

^j^ Anesthesia and Intensive Care for Adults, Fondazione IRCCS Ca' Granda, Ospedale Maggiore Policlinico, Milan, Italy

^k^ Department of Thoracic Surgery, Fondazione IRCCS Ca' Granda Ospedale Maggiore Policlinico, Milan, Italy

**Additional Methods:**

**Perfusion solution:**

The present protocol used an acellular perfusion fluid with extra-cellular electrolyte composition. The solution was freshly prepared in sterile conditions in a laminar flow hood, using: albumin, NaCl, Perfadex (XVIVO Perfusion AB, Göteborg, Sweden), amphotericin B, glucose, CaCl_2_, KCl, K_3_PO_4_, MgSO_4_, and heparin. Although two separate lots of albumin with different sodium concentrations were used, electrolyte composition was maintained stable. Characteristics of the two solutions were: osmolality 294 and 286 mOsm/kg; albumin 8.8 and 8.9 g/dl; total protein 9.4 g/ dl for both lots. The perfusion fluid was filtered using a vacuum filtration unit to remove any particulate impurity or bacterial contamination and stored for a maximum of 5 days to avoid bacterial growth. Before each experiment, the fluid was added with cefazolin and NaHCO_3_, whereas no treatment with corticosteroids or other anti-inflammatory drugs was performed.

**Additional Results:**

**Timing of procedures during the experiment**

| **Procedures** | | **Time (min)** |
| --- | --- | --- |
| **Harvest Total** |  | 99 [94 - 110 ] |
|  | **Harvest preflush** | 75 [66 - 85 ] |
|  | **Harvest postflush** | 26 [25 - 27 ] |
| **Cold ischemia Total** |  | 184 [167 - 187 ] |
|  | **Flushing** | 12 [11 - 12 ] |
|  | **Fridge** | 65 [61 - 70 ] |
|  | **Back Table** | 76 [74 - 96 ] |

Table S1: Timing of procedures during the experiments. Data are reported as median and [IQR].

**PaO_2_/FiO_2_ and Shunt fraction (Qs/Qt %) during the experiment**

Table S2: PaO_2_/FiO_2_ ratio (mmHg) [upper panel] and Qs/Qt % [lower panel] in blood samples from left atrium and each pulmonary vein throughout the different phases of the experiment. One Way ANOVA, p<0.05 as significant, * vs Baseline, # vs Left Inferior Bronchial Occlusion, ^ vs Left Superior Bronchial Occlusion.

|  |  | **Baseline** | **Left Inferior Bronchial Occlusion** | **Left Superior Bronchial Occlusion** | **p-value** |
| --- | --- | --- | --- | --- | --- |
| **PaO_2_/FiO_2_, mmHg** |  |  |  |  |  |
|  | **Left Atrium** | 439 [383 - 449] | 299 [188 - 373]** | 349 [347 - 377]* | 0.0051 |
|  | **Right Superior Vein** | 468 [454 - 505] | 472 [455 - 484] | 419 [402 - 436] | 0.1315 |
|  | **Right Inferior Vein** | 392 [344 - 468] | 319 [307 - 356] | 334 [301 - 395] | 0.1845 |
|  | **Left Superior Vein** | 426 [416 - 449] | 407 [353 - 472] | 45 [43 - 67]**## | 0.0015 |
|  | **Left Inferior Vein** | 363 [258 - 404] | 47 [46 - 54]***^^ | 350 [214 - 399] | 0.0008 |
| **Qs/Qt, %** |  |  |  |  |  |
|  | **Left Atrium** | 23 [20 - 27] | 30 [29 - 42] | 30 [27 - 30] | 0.0799 |
|  | **Right Superior Vein** | 18 [17 - 20] | 21 [16 - 21] | 21 [21 - 26] | 0.0584 |
|  | **Right Inferior Vein** | 23 [20 - 30] | 26 [25 - 34] | 28 [28 - 30] | 0.3508 |
|  | **Left Superior Vein** | 23 [22 - 24] | 27 [17 - 33] | 97 [78 - 106]**## | 0.0011 |
|  | **Left Inferior Vein** | 29 [25 - 38] | 97 [88 - 103]**^^ | 30 [23 - 48] | 0.0011 |

Table S2: PaO_2_/FiO_2_ ratio (mmHg) [upper panel] and Qs/Qt % [lower panel] in blood samples from left atrium and each pulmonary vein throughout the different phases of the experiment. * p<0.05 vs BASELINE; ** p<0.01 vs BASELINE; *** p<0.001 vs BASELINE; # p<0.05 vs Left Inferior Bronchial Occlusion; ## p<0.01 vs Left Inferior Bronchial Occlusion; ^ p<0.05 vs Left Superior Bronchial Occlusion; ^^ p<0.01 vs Left Superior Bronchial Occlusion

**Quantitative CT scan data at baseline and during the experiment**

| **A) Total Lung** | **Baseline** | **Left Inf Br Occlusion** | **Left Sup Br Occlusion** | **p-value** | **B) Right Lung** | **Baseline** | **Left Inf Br Occlusion** | **Left Sup Br Occlusion** | **p-value** |
| --- | --- | --- | --- | --- | --- | --- | --- | --- | --- |
| **Air volume, mL** | 1169.9 [1165.1 - 1281.2] | 836.6 [728.8 - 1051.3]** | 880.8 [852.7 - 1011.5]** | 0.001 | **Air volume, mL** | 738 [705.1 - 863.5] | 671.7 [558.4 - 772.5] | 678.1 [671.1 - 697.3] | 0.219 |
| **Tissue weight, g** | 555.2 [435.2 - 568.5] | 600 [459 - 625.9] | 641.5 [503.6 - 698.7] | 0.079 | **Tissue weight, g** | 308.2 [246.3 - 310.7] | 340.7 [248.5 - 375.7] | 377.5 [263.9 - 383]** | 0.006 |
| **Over-aerated, %** | 0.8 [0.8 - 1.2] | 0.6 [0.5 - 0.8] | 0.4 [0.4 - 0.4]* | 0.016 | **Over-aerated, %** | 1.3 [0.8 - 1.4] | 0.7 [0.7 - 1.1] | 0.6 [0.6 - 0.7] | 0.058 |
| **Normally-aerated, %** | 68.2 [64.2 - 72.6] | 45.9 [41.5 - 47.2]* | 39.8 [35.2 - 45.2]** | 0.007 | **Normally-aerated, %** | 75.4 [72.5 - 79.2] | 62.2 [59.6 - 62.7]* | 53.9 [48.8 - 65.5]* | 0.009 |
| **Poorly-aerated, %** | 22 [19 - 26] | 38.6 [35.5 - 38.6]** | 37 [35.5 - 41.1]** | 0.003 | **Poorly-aerated, %** | 18.8 [17.2 - 20.9] | 27.6 [27.3 - 31.4]** | 29.8 [25.3 - 30.7]** | 0.003 |
| **Non-aerated, %** | 8.9 [7.3 - 9.3] | 16.5 [14.7 - 19]* | 18.9 [18.7 - 22.7]** | 0.006 | **Non-aerated, %** | 4.5 [3.2 - 6] | 8.3 [5.2 - 8.4] | 15.9 [8 - 16.2] | 0.078 |
| **C) Left Superior Lung** | **Baseline** | **Left Inf Br Occlusion** | **Left Sup Br Occlusion** | **p-value** | **D) Left Inferior Lung** | **Baseline** | **Left Inf Br Occlusion** | **Left Sup Br Occlusion** | **p-value** |
| **Air volume, mL** | 188.4 [181.1 - 191.4] | 119.7 [113.6 - 192] | 32.4 [27.8 - 36.3]**§ | 0.003 | **Air volume, mL** | 251.9 [243.4 - 268.6] | 55.7 [50.7 - 73.8]*** | 178.3 [153.9 - 224.7]§ | 0.001 |
| **Tissue weight, g** | 72.8 [61.1 - 80.5] | 66.6 [53.1 - 88] | 75.7 [70.7 - 81.1] | 0.443 | **Tissue weight, g** | 155.4 [148 - 166.7] | 163 [159.8 - 192.7] | 187.8 [158.6 - 245.5] | 0.122 |
| **Over-aerated, %** | 0.9 [0.6 - 2.1] | 0.9 [0.7 - 1] | 0 [0 - 0.1]**§§ | 0.003 | **Over-aerated, %** | 0.3 [0.2 - 0.6] | 0 [0 - 0.1]** | 0.2 [0.1 - 0.2]§ | 0.003 |
| **Normally-aerated, %** | 72 [66.5 - 76.3] | 63.2 [57.7 - 67.9] | 6.6 [3.3 - 11.1]***§§ | 0.001 | **Normally-aerated, %** | 57.7 [47.3 - 62.6] | 4.7 [3.9 - 7.2]*** | 23.7 [23.5 - 29.9]§§ | 0.000 |
| **Poorly-aerated, %** | 17.8 [15 - 21] | 24.5 [24 - 30.6]* | 62.1 [47 - 65]***§§ | 0.000 | **Poorly-aerated, %** | 28.5 [24.1 - 34.2] | 57.7 [55.7 - 57.9]*** | 41.9 [38.9 - 48.4]§ | 0.001 |
| **Non-aerated, %** | 7.7 [5.5 - 10.9] | 10.7 [7.3 - 11.4] | 27.3 [26.8 - 28.3]**§§ | 0.002 | **Non-aerated, %** | 13.3 [13 - 17.5] | 37.6 [34.8 - 40.4]** | 28.1 [21 - 31.2] | 0.005 |

Table S3: Quantitative CT scan data at baseline, after left inferior bronchial (Inf Br) occlusion and after superior bronchial (Sup Br): Overall analysis of total lung and selective analysis of right, left superior and left inferior lung. Over-aerated: -1000 -901 HU, Hounsfield Unit; Normally-aerated -900 -501 HU; Poorly-aerated -500 -101 HU; Non-aerated -100 +200 HU. Data are expressed as mean±SD, * p<0.05 vs Baseline, ** p<0.01 vs Baseline, *** p<0.001 vs Baseline, § p<0.05 vs Left Inferior Bronchial Occlusion, §§ p<0.01 vs Left Inferior Bronchial Occlusion, §§§ p<0.001 vs vs Left Inferior Bronchial Occlusion. Air volume, tissue weight and density distribution were compared with proc mixed on ranks on timing.

**Histological findings at the end of the experiment in the different lobes.**

|  | **Right Apex** | **Right Medium** | **Right Base** | **Left Apex** | **Left Medium** | **Left Base** | **p-value** |
| --- | --- | --- | --- | --- | --- | --- | --- |
| **Emphysematous change** | 1 [1 - 1] | 1 [1 - 1] | 1 [1 - 1] | 1 [1 - 1] | 1 [1 - 1] | 1 [1 - 1] | Stopped because of infinite likelihood |
| **Interstitial congestion** | 1 [0 - 1] | 1 [1 - 1] | 3 [1 - 3] | 2 [2 - 2] | 2 [1 - 2] | 3 [2 - 3] | 0.0449 |
| **Alveolar hemorrhage** | 0 [0 - 1] | 0 [0 - 1] | 3 [0 - 3] | 2 [2 - 2] | 1 [1 - 2] | 3 [3 - 3]* | 0.0211 |
| **Alveolar neutrophil infiltration** | 1 [1 - 1] | 2 [1 - 2] | 1 [1 - 1] | 1 [1 - 2] | 2 [1 - 2] | 2 [1 - 2] | 0.2485 |
| **Alveolar macrophage proliferation** | 2 [2 - 2] | 2 [2 - 2] | 2 [2 - 3] | 2 [2 - 3] | 2 [2 - 2] | 3 [2 - 3] | 0.0894 |
| **Alveolar type II pneumocytres proliferation** | 1 [0 - 1] | 1 [1 - 1] | 1 [0 - 1] | 1 [1 - 1] | 1 [1 - 1] | 1 [1 - 1] | 0.229 |
| **Interstitial lymphocytes proliferation** | 2 [1 - 2] | 2 [2 - 2] | 2 [2 - 2] | 2 [2 - 2] | 2 [2 - 2] | 2 [2 - 2] | 0.4031 |
| **Interstitial thickening** | 1 [1 - 1] | 1 [1 - 1] | 1 [1 - 1] | 1 [1 - 1] | 1 [1 - 1] | 1 [1 - 1] | 0.2721 |
| **Hyaline membrane formation** | 1 [0 - 1] | 1 [1 - 1] | 1 [1 - 1] | 1 [1 - 1] | 1 [1 - 1] | 1 [1 - 1] | 0.3866 |
| **Interstitial fibrosis** | 0 [0 - 0] | 0 [0 - 0] | 0 [0 - 0] | 0 [0 - 0] | 0 [0 - 0] | 0 [0 - 0] | 0.5627 |
| **Organization of alveolar exudate** | 0 [0 - 0] | 0 [0 - 0] | 0 [0 - 0] | 0 [0 - 0] | 0 [0 - 0] | 0 [0 - 0] | 0.443 |
| **Total** | 10 [7 - 10] | 11 [10 - 11] | 13 [10 - 15] | 14 [13 - 16] | 13 [13 - 14] | 16 [15 - 17]*^ | 0.0211 |

Table S4: Histological findings at the end of the experiment in the different lobes. Each pathological change in all lung lobes studied of every experiment was evaluated with a score ranging 0-3 and indicating different degree of damage: 0, absence; 1, mild; 2, moderate; 3, severe. Proc mixed n ranks * p<0.05 vs right apex lobe; ^ p<0.05 vs right middle lobe.
